# Molecular detection of *Rickettsia felis* in dogs, rodents and cat fleas in Zambia

**DOI:** 10.1186/s13071-019-3435-6

**Published:** 2019-04-11

**Authors:** Lavel Chinyama Moonga, Kyoko Hayashida, Ryo Nakao, Malimba Lisulo, Chiho Kaneko, Ichiro Nakamura, Yuki Eshita, Aaron S. Mweene, Boniface Namangala, Chihiro Sugimoto, Junya Yamagishi

**Affiliations:** 1 Division of Collaboration and Education, Research Center for Zoonosis Control, Hokkaido University, Kita 20, Nishi 10, Kita-Ku, Sapporo, Hokkaido 001-0020 Japan; 2 Laboratory of Parasitology, Department of Disease Control, Graduate School of Veterinary Medicine, Hokkaido University, Kita 18, Nishi 9, Kita-Ku, Sapporo, Hokkaido 060-0818 Japan; 3 The University of Edinburgh, College of Medicine and Veterinary Medicine, Deanery of Biomedical Sciences, 1 George Square, Edinburgh, EH8 9JZ UK; 4 Project for Zoonoses Education and Research, Faculty of Agriculture, University of Miyazaki, 1-1, Gakuen-kibanadai-nishi, Miyazaki, 889-2192 Japan; 5 Unit of International Cooperation, Research Center for Zoonosis Control, Hokkaido University, Kita 20, Nishi 10, Kita-Ku, Sapporo, Hokkaido 001-0020 Japan; 6 Department of Disease Control, School of Veterinary Medicine, University of Zambia, P. O. Box 32379, Lusaka, Zambia; 7 Department of Paraclinical Studies, School of Veterinary Medicine, University of Zambia, PO Box 32379, Lusaka, Zambia; 8 African Centre of Excellence for Infectious Diseases of Human and Animals, University of Zambia, PO Box 32379, Lusaka, Zambia; 9 Global Station for Zoonosis Control, GI-CoRE, Hokkaido University, Sapporo, Hokkaido Japan

**Keywords:** *Rickettsia felis*, Cat flea, Dogs, Rodents, Zoonosis, Zambia

## Abstract

**Background:**

Flea-borne spotted fever is a zoonosis caused by *Rickettsia felis*, a Gram-negative obligate intracellular bacterium. The disease has a worldwide distribution including western and eastern sub-Saharan Africa where it is associated with febrile illness in humans. However, epidemiology and the public health risks it poses remain neglected especially in developing countries including Zambia. While *Ctenocephalides felis* (cat fleas) has been suggested to be the main vector, other arthropods including mosquitoes have been implicated in transmission and maintenance of the pathogen; however, their role in the epidemiological cycle remains to be elucidated. Thus, the aim of this study was to detect and characterize *R. felis* from animal hosts and blood-sucking arthropod vectors in Zambia.

**Methods:**

Dog blood and rodent tissue samples as well as cat fleas and mosquitoes were collected from various areas in Zambia. DNA was extracted and screened by polymerase chain reaction (PCR) targeting genus *Rickettsia* and amplicons subjected to sequence analysis. Positive samples were further subjected to *R. felis*-specific real-time quantitative polymerase chain reactions.

**Results:**

*Rickettsia felis* was detected in 4.7% (7/150) of dog blood samples and in 11.3% (12/106) of rodent tissue samples tested by PCR; this species was also detected in 3.7% (2/53) of cat fleas infesting dogs, co-infected with *Rickettsia asembonensis*. Furthermore, 37.7% (20/53) of cat flea samples tested positive for *R. asembonensis*, a member of spotted fever group rickettsiae of unknown pathogenicity. All the mosquitoes tested (*n* = 190 pools) were negative for *Rickettsia* spp.

**Conclusions:**

These observations suggest that *R. felis* is circulating among domestic dogs and cat fleas as well as rodents in Zambia, posing a potential public health risk to humans. This is because *R. felis*, a known human pathogen is present in hosts and vectors sharing habitat with humans.

**Electronic supplementary material:**

The online version of this article (10.1186/s13071-019-3435-6) contains supplementary material, which is available to authorized users.

## Background

*Rickettsia felis* is an obligate intracellular gram-negative alpha-proteobacterium that causes zoonotic cat flea typhus also known as flea-borne spotted fever in humans. The bacterium was first described by Adams et al. in 1990 in the cytoplasm of midgut cells of the cat flea *Ctenocephalides felis* [1]. It was later characterized as *R. felis* and confirmed as the causative agent of flea-borne spotted fever in humans [2, 3]. The genus *Rickettsia* is divided into three groups based on genotyping: the ancestral group, the typhus group and the spotted fever group to which *R. felis* belongs [4].

Spotted fever rickettsioses are one of the emerging infectious diseases to which little attention has been accorded. The first human case of *R. felis* infection was reported in 1994 in Texas, USA [3]. Since then, it remained neglected until its emergence as a cause of febrile illness in sub-Saharan Africa [5]. It has since been reported in eastern and western sub-Saharan Africa [6, 7] and North Africa [8]. *Rickettsia felis* infections in humans have also been reported in the USA, Brazil and Mexico in the Americas with varying clinical signs and severity, in Taiwan, Thailand, South Korea, France, Germany, Spain and other parts of the world [8, 9]. This worldwide distribution of *R. felis* infections in humans resulted into its consideration as a global emerging threat to human health [9, 10]. Once a susceptible human is infected, rickettsiae mainly invade endothelial cells and cause vasculitis, acute flu-like symptoms, fever, chills, headache, skin rash and photophobia [11] as the clinical manifestations of flea-borne spotted fever.

Cat fleas have been considered as the hosts and vectors of *R. felis* [8]. However, recent reports have suggested other arthropods such as other flea species [12], ticks [13], mites [14], and booklice [15] as potential vectors in the epidemiological cycle through detection of *R. felis* using molecular techniques. *Rickettsia felis* has also been detected in *Aedes albopictus* in Gabon [16] and was experimentally proven to be transmitted by *Anopheles gambiae* in a mouse model [17]. This claim was further strengthened by the elucidation of co-infection with malaria and *R. felis* among febrile patients in malaria-endemic areas of Senegal, suggesting co-transmission by mosquitoes [7].

Domestic dogs and cats are considered mammalian reservoir hosts for *R. felis* [18, 19]. Other proposed mammalian reservoirs include domestic/peridomestic rodents, opossums and raccoons which are documented to maintain the pathogen in peridomestic areas [20, 21]. However, what is unclear is the interaction between the domestic/peridomestic cycles for pathogen transmission and maintenance in nature. Cat fleas may play an essential role in both transmission cycles resulting in potential risk of human exposure due to their indiscriminate feeding behavior [22]. Additionally, *R. felis* can be circulating in cat fleas *via* transstadial and transovarial transmission consequently fleas acting as reservoirs and vectors. Therefore, understanding the host-vector relationship and epidemiology of *R. felis* is vital for pathogen surveillance and effective control measures in case of outbreaks as these animals share habitat with humans.

Although *R. felis* is frequently detected in western and eastern sub-Saharan Africa, little is known about its epidemiology and the public health risk it poses in Zambia. On the other hand, other spotted fever group rickettsiae have been reported from Zambia. Tamaki et al. [23] reported 16.7% seroprevalence for antibodies against *Rickettsia conorii* in humans despite cross-reactivity of spotted fever group rickettsia and *R. felis* on serology having been documented [24], hence possibility of misdiagnosing some closely related species cannot be excluded. Later Nakayima et al. [25] found molecular evidence of spotted fever group rickettsiae closely related to *Rickettsia africae* (the causative agent for African tick bite fever) in free-ranging non-human primates in Mambwe District near Luangwa National Park, Zambia. With the endemic cases of malaria [26] little attention has been given to other pathogens that cause febrile illness, hence possibilities of misdiagnosis could be high. A study in Kenya found strong evidence of the association of *R. felis* with a fever of unknown origin [27].

To date, no studies have been carried out to elucidate the prevalence of *R. felis* in animals, humans and arthropods in Zambia. Hence, it is of prime importance to understand the epidemiology of *R. felis* both in mammalian hosts and arthropod vectors/potential vectors as well as the risk of human transmission. The aim of this study was thus to detect and characterize *R. felis* from animal hosts and possible arthropod vectors in Zambia. The findings would provide the baseline for future surveillance of *Rickettsia* spp. in Zambia and risk assessment of human exposure to the infection.

## Methods

### Sample collection

**Fig. 1:**
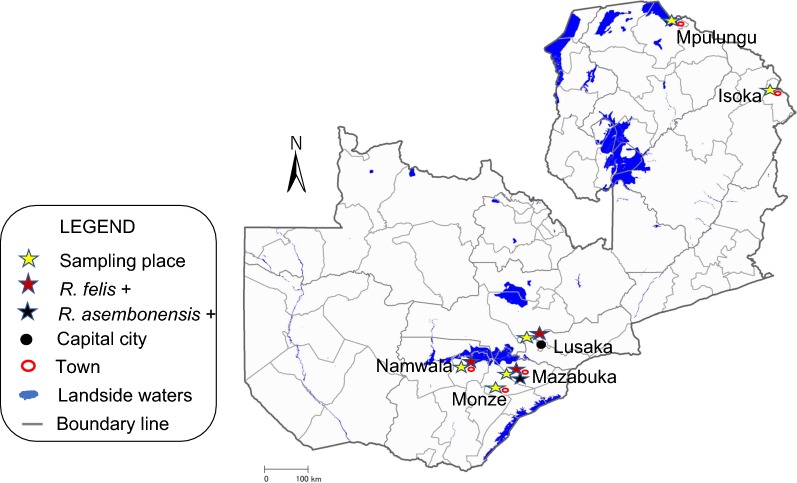
Map of Zambia showing sample collection sites and associated *Rickettsia* species detected. The map was downloaded from the International Steering Committee for Global Mapping

Blood samples from visibly healthy domestic dogs were collected from Lusaka District in Lusaka Province, and from Mazabuka and Monze districts in the Southern Province of Zambia (Fig. 1) in 2015. Genomic DNA was extracted from blood using the DNAzol kit (Molecular Research Center, Cincinnati, OH) according to manufacturers’ protocols. Genomic DNA samples (*n* = 106) from rodents were collected in a previous study [28]. The rodent species were identified by partial sequence of the cytochrome *c* oxidase subunit 1 gene (*cox*1) or cytochrome *b* gene (*cytb*). The analyzed species included *Mastomys* sp., *Steatomys* sp., *Lemniscomys* sp., *Saccostomus* sp., *Rattus rattus* and the gerbil.

Cat flea (*Ctenocephalides felis*) samples (*n* = 53) infesting different dogs in Mazabuka District, Southern Province, were collected in 2016 and identified to species level using morphological characteristics [29]. After identification to the species level, each cat flea was separately homogenized and DNA was extracted. Mosquitoes of different species were collected by using the CDC and BG traps in Zambia during 2016–2017. They were then identified to the species level using morphological taxonomic keys. The mosquitoes were then pooled (*n* ≤ 10) based on sex and species, homogenized and DNA extracted using Takara Simpleprep kit (Takara, Shiga, Japan).

### PCR amplification and amplicon sequencing

Conventional PCR targeting the partial *gltA* gene for genus *Rickettsia* was essentially performed using Ampdirect plus buffer (Shimadzu, Tokyo, Japan) with BioTaq Polymerase (Bioline, London, UK) as described by manufacturers. The PCR was performed at 94 °C DNA denaturation, 54 °C annealing and 72 °C extension temperatures for 35 cycles using the primers described by Gaowa et al. [30]. The amplicons were resolved on a 1.2% agarose gel stained with GelRed (Biotium, Hayward, CA) and visualized under UV light. Positive samples with *gltA* PCR were processed for PCR targeting the outer membrane protein A (*OmpA*) and outer membrane protein *B* (*OmpB*) genes using primers designed in this study. The *OmpA* and *OmpB* gene fragment amplification primers were designed using the Primer-blast tool in NCBI (https://www.ncbi.nlm.nih.gov/tools/primer-blast/). The *OmpA* and *OmpB* gene sequences for *R. felis* accession number CP000053 retrieved from GenBank were used as references for primer design. The PCRs were essentially conducted as described above with modification of annealing temperature (48 °C for the *OmpA* and *OmpB*) and amplicons were electrophoresed on 2% and 1.2% agarose gels respectively. *Rickettsia monacensis* DNA extracted from cell culture was used as a positive control to avoid sample contamination by *R. felis* DNA from the positive control and nuclease-free water was used as a negative control in all assays. All samples that were *gltA* PCR-positive were further subjected to partially *R. felis-*specific qPCR targeting biotin synthase gene (*bioB*) and a *R. felis* unique segment of the outer membrane protein gene (*RfelB*) using primers and probe described previously [31, 32]. All primers and probes used are shown in Table 1.

**Table 1:** Primer sets used for PCR amplification and sequencing

Target gene	Oligonucleotide sequence (5′–3′)	Specificity	Tm (°C)	Size (bp)	References
*gltA*	F-CGAACTTACCGCTATTAGAATG^a^	*Rickettsia* genus-specific	54	581	[30]
	R-CTTTAAGAGCGATAGCTTCAAG^a^				
*OmpA*	F-TGCAGGGCTTAGATATTCGGC^a^	Spotted fever group-specific	48	258	This study
	R-AAGCTGTTGGTAAAGGAGCA^a^				
*OmpB*	F-GGACCTGAAGCTGGAGCAAT^a^	*Rickettsia* genus-specific	48	776	This study
	R-CTGTCAGGCTGGCTGATGAA^a^				
*bioB*	F-ATGTTCGGGCTTCCGGTATG	*R. felis*-/ *R. asembonensis*-specific	60	120	[31]
	R-CCGATTCAGCAGGTTCTTCAA				
	Probe 6FAM-GCTGCGGCGGTATTTTAGGAATGGG-TAMRA				
*RfelB*	F-TAATTTTAACGGAACAGACGGT	*R. felis-*specific	60	97	[32]
	R-GCCTAAACTTCCTGTAACATTAAAG				
	Probe FAM-TGCTGCTGGTGGCGGTGCTA-BHQ				

^a^Primer used for PCR and sequencing

*Abbreviations*: Tm, annealing temperature; size, amplicon product size, F, forward primer; R, reverse primer

PCR products were purified using ExoSap-IT (Thermo Fisher Scientific, Waltham, MA) according to the manufacturer’s instructions. DNA concentration was measured using a Nanodrop spectrophotometer (Thermo Fisher Scientific). Amplicons were cycle-sequenced by BigDye Terminator v3.1 cycle sequencing kit (Thermo Fisher Scientific) with the same primers as used for PCR amplification and analyzed on a 3500 × l Genetic Analyzer (Applied Biosystems, Foster City, CA). The DNA sequences obtained were submitted to the DNA Data Bank of Japan (DDBJ) (http://www.ddbj.nig.ac.jp) under the accession numbers LC431490–LC431502.

### Alignment and phylogenetic analysis

The newly generated nucleotide sequences were assembled and aligned with relevant sequences from GenBank in NCBI (http://blast.ncbi.nlm.nih.gov/Blast.cgi) using ClustalW aligner [33]. Phylogenetic analysis of sequences obtained in the present study along with reference sequences was performed by Neighbor-Joining method. Bootstrap support was based on 500 replicates. Alignment and phylogenetic analyses were conducted in MEGA 7.0.2.1 software [34].

### Statistics

Statistical analysis was performed by the Chi-square statistical test to determine the statistical difference in *Rickettsia* sp. prevalence among dogs from densely populated urban areas of Lusaka District and sparsely populated rural areas of Monze and Mazabuka Districts. Differences were considered significant at *P *≤ 0.05.

## Results

A sample was considered positive to the species level upon being positive for at least two markers (Additional file 1: Table S1). Based on this criterion, a sample was considered *R. felis-*positive when the partial *gltA* gene fragment nucleotide have homology of more than 99% to *R. felis* strain URRXCal2 (GenBank: CP000053) reference sequence [4] and, partially *R. felis-*specific *bioB* or *R. felis* unique fragment of outer membrane protein B (*RfelB*) qPCR was positive. Furthermore, samples positive for both qPCR markers were considered to be positive for *R. felis*. Sequence homology based on outer membrane protein A (*OmpA*) and outer membrane protein B (*OmpB*) genes was used to further confirm other *Rickettsia* species including *Rickettsia felis*-like organisms (RFLOs) to the species level. Isolates not meeting above criteria were identified to the spotted fever group level.

*Rickettsia felis* was detected in 4.7% (7/150) of the dogs from Lusaka and Mazabuka, and 3.7% (2/53) of the cat fleas collected from Mazabuka. Furthermore, 11.3% (12/106) of the rodents from Lusaka and Namwala were also positive for *R. felis* (Table 2). All the positive rodents were of the *Mastomys* sp. The obtained sequences of *gltA* gene fragment from dog and rodent samples were 99–100% similar to *R. felis* strain URRXCal2 (GenBank: CP000053) and further confirmed by *R. felis* specific qPCR (Additional file 1: Table S1). All dog blood samples from Monze (*n* = 50) were negative for *Rickettsia* spp.

**Table 2:** Prevalence of spotted fever group rickettsiae and their hosts in Zambia

Host	Locality	No. of samples	No. positive for *R. asembonensis* only (%)	No. positive for *R. felis* only (%)	No. positive for *R. felis* and *R. asembonensis* (%)
Cat fleas (*C. felis*)	Mazabuka	53	20 (37.7)	0	2 (3.7)
Domestic dogs	Lusaka	50	0	6 (12.0)	0
	Mazabuka	50	0	1 (2.0)	0
	Monze	50	0	0	0
*Mastomys* sp.	Lusaka	33	0	2 (6.1)	0
	Namwala	44	0	10 (22.7)	0
Gerbils	Namwala	13	0	0	0
*Steatomys* sp.	Namwala	10	0	0	0
*Saccostomus* sp.	Namwala	3	0	0	0
*Rattus rattus*	Namwala	3	0	0	0
Mosquitoes (*Culex* spp., *Aedes* spp., *Anopheles* spp.)^a^	Lusaka	154	0	0	0
	Mpulungu	33	0	0	0
	Isoka	3	0	0	0
Total			20	19	2

^a^See Additional file 1: Table S2 for details

Among the analyzed cat flea samples obtained from Mazabuka, 3.7% (2/53) were positive for *R. felis-*specific *RfelB* qPCR (Table 2, Additional file 1: Table S1). Interestingly, their *gltA* sequences showed higher homology to *R. asembonensis* than *R. felis* suggesting that these cat fleas were co-infected with *R. felis* and *R. asembonensis*. The qPCRs targeting *bioB* (mean Ct = 26) which is specific for both *R. felis* and *R. asembonensis* were also positive, but their Ct values were lower than those for *RfelB* (mean Ct = 36). The lower *Ct* values of *bioB* qPCR as compared to *RfelB* suggests that the population of *R. asembonensis* was higher than that of *R. felis* in the cat flea samples. It is also consistent with the observed *R. asembonensis* type *gltA* sequences. The presence of *R. asembonensis* was further confirmed by sequence homology based on the *OmpA* and *OmpB* gene fragments. The sequences of the *gltA*, *OmpA* and *OmpB* gene fragments obtained from cat fleas were 100%, 99% and 100% similar to *Rickettsia asembonensis* sequences with accession numbers JN315968, KY650698 and KY650699, respectively. The overall positive rate for *R*. *asembonensis* in Mazabuka cat fleas was 37.7% (20/53).

**Fig. 2:**
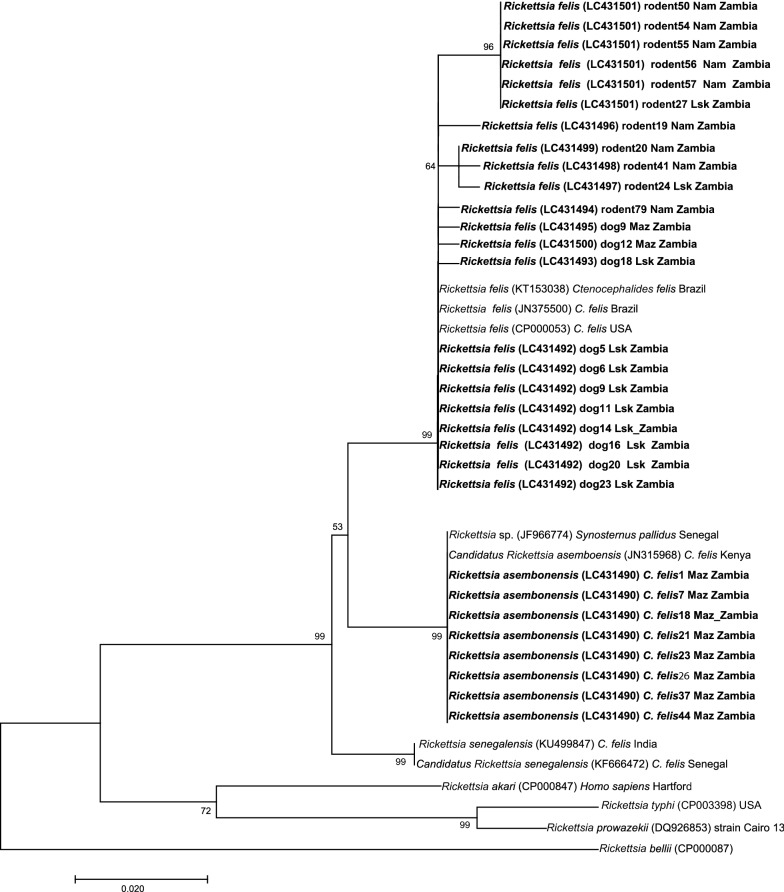
Unrooted phylogenetic tree based on sequences of the *gltA* gene. Neighbor-joining phylogenetic relationships of spotted fever group rickettsiae detected in cat fleas, dogs and rodents in Zambia. The tree was based on 321 bp fragment of *gltA* gene. Bold labels represent sequences from this study with the detected species name, accession number, and host and place of collection in the label

As shown in Fig. 2, the *gltA* sequences from dog blood and rodent tissue samples clustered with *R. felis* in the phylogenetic analysis regardless of the sampling location. In contrast, those of cat flea samples were separated from *R. felis* sequences and clustered with *R. asembonensis*. The *gltA* gene sequences obtained from rodent samples were more diversified from the reference sequences previously identified in cat fleas (Fig. 2). Further phylogenetic analysis based on the *rOmpB* gene fragment from cat flea samples similarly clustered with *R. asembonensis* (data not shown).

One hundred ninety of pooled samples from different mosquito species, *Culex* spp., *Aedes* spp. and *Anopheles* spp. (listed in Additional file 1: Table S2) collected from Lusaka, Mpulungu and Isoka districts were all negative for *Rickettsia* spp. by *gltA* PCR. Among the analyzed mosquito samples, 81.1% (154/190) were collected from Lusaka District, the same location where *R. felis* was detected in dogs and rodents. Among the sampled mosquitoes from Lusaka District, 87% (134/154) belonged to *Culex* spp. with the others being *Anopheles* spp. and *Aedes* spp. In contrast to Lusaka District, 87.8% (29/33) of the samples from Mpulungu District were *Aedes* spp. (Additional file 1: Table S2) suggesting that *Culex* spp. are more predominant in the urban Lusaka township.

## Discussion

Investigation of neglected zoonotic pathogens in domestic pets and vectors infesting them is important in the control and eradication of zoonotic diseases. In this study, we found 4.7% (7/150) prevalence of *R. felis* in domestic dogs. The detected genotype is identical to reported one found in GenBank which was isolated from cat fleas and mosquitoes, and is known to cause flea-borne spotted fever in humans [5, 35]. This highlights the significance of domestic dogs as potential reservoirs of *R. felis*. It is therefore important for dog owners to be aware of rickettsia infection risk as concurrent *R. felis* infections in a positive dog and its infected owners have already been reported [36].

Among the blood samples from dogs in Lusaka, Mazabuka and Monze districts, it was found that dogs from the urban areas of Lusaka have a significantly higher prevalence of *Rickettsia* sp. (40%, 20/50) than dogs from the rural areas of Mazabuka (10%, 5/50) and Monze (0%, 0/50). This could be due to the off-host nature of fleas resulting in feeding on many animals in close proximity. The high dog population density and closeness of households in the urban areas of Zambia could possibly make it easy for cat fleas to feed on many dogs within a close proximity. Therefore, the host diversity of cat fleas [37] in different demographical setups could help explain the differences in prevalences of *Rickettsia* sp. in dogs from rural and urban areas.

It is known that the cat flea is a vector for flea-borne spotted fever causative agent. In this study, *R. felis* was also detected in cat fleas in the same sampling place (Mazabuka District) where dogs were positive for *R. felis*. This molecular-based evidence implies that *R. felis* is circulating with domestic dogs serving as reservoirs and cat fleas as vectors in Zambia. This is of public health significance as the bacterium is potentially maintained among domestic dogs and cat fleas as earlier reported [38]. This results in a potential risk of human infections through cat flea bites as they feed indiscriminately, hence effective vector-borne infection control measures are necessary to prevent zoonotic disease outbreaks.

High prevalence of *R. asembonensis* (41.5%) was also observed with low prevalence of *R. felis* (3.7%) in cat fleas. This result is in agreement with previous studies reporting up to 100% prevalence of *R. asembonensis* in cat fleas with low *R. felis* prevalence [37, 39–44]. Interestingly, two of the cat flea samples positive for *R. felis* were also positive for *R. asembonensis.* These findings provide evidence for co-infection of *R. felis* and *R. asembonensis* in cat fleas. Furthermore, it is important to investigate the pathogenicity of *R. asembonensis* to understand its infectivity in dogs and more importantly humans [45, 47]. This is because cat fleas feed indiscriminately, hence the potential risk of human exposure through flea bites.

This study also presents molecular evidence of *R. felis* in rodents *Mastomys* sp. in Zambia, suggesting that the latter could be potential reservoirs of *R. felis*. Rodents have been implicated as reservoir hosts for diverse zoonotic pathogens ranging from viruses, protozoans, fungi, helminths to bacteria [46] including *Rickettsia* spp. [20]. Limited studies have been conducted to understand the role of rodents in the epidemiology and ecology of *R. felis* [20, 42, 47, 48], despite many studies having detected *R. felis* DNA in fleas infesting rodents [8, 42, 43, 49]. This result adds *Mastomys* sp. to the rodent species implicated to be potentially maintaining *R. felis* in nature. Rodents are suggested to be vital for maintenance of *R. felis* resulting in sporadic cases of rickettsioses in humans as they share habitat with humans and dogs in the presence of fleas as vectors [50]. Therefore, interplay among mammalian hosts, fleas and humans need to be clarified in order to understand the zoonosis transmission cycle and possible control measures.

Many other blood-sucking arthropods with the potential to transmit rickettsiae are present in Zambia. Therefore, surveillance for neglected vector-borne zoonoses such as spotted fever rickettsioses posing a potential emerging public health threat is necessary. It is for this reason that different mosquito species, including *Culex* spp., *Aedes* spp. and *Anopheles* spp. (Additional file 1: Table S2) were screened for *Rickettsia* spp. and were notably all negative. This suggests that mosquitos may not be playing an essential role in the epidemiology of *R. felis* in Zambia despite earlier reports [7, 35]. The role of these mosquito species in *R. felis* epidemiology is still unclear despite recent advances in transmission potential studies, hence further studies are needed [7, 16, 17].

## Conclusions

This study provides, to our knowledge for the first time in Zambia, evidence of the occurrence of *R. felis* in domestic dogs, rodents and cat fleas. In particular, the genotype identified from the dogs was identical to a reported genotype detected in cat fleas. Thus, we surmise the possibility of involvement of dogs and cat fleas in the maintenance and transmission of *R. felis* in Zambia. Besides, the potential of rodents as reservoirs is not negligible even though observed genotypes in *Mastomys* sp. were slightly diversified from the reported genotype in cat fleas. Interestingly, cat fleas were coinfected with *R. asembonensis* of unknown pathogenicity with a potential to infect humans. These findings provide evidence of potential circulation of *R. felis* in Zambia and a risk of human infection. Further studies of potential *R. felis* infections in humans are needed to understand its epidemiology and ecology.

## Additional file

**Additional file 1: Table S1.** Criteria for *Rickettsia* species detection and annotation. **Table S2.** Detailed description of mosquito species sampled and analyzed, and sampling places in Zambia.

## Abbreviations

qPCR: quantitative real-time PCR
Ct: Cycle threshold
PCR: Polymerase chain reaction
*gltA*: Citrate synthetase gene
*OmpA*: Outer membrane protein A gene
*OmpB*: Outer membrane protein B gene
*bioB*: Biotin synthase gene
*RfelB*: *R. felis* unique segment of *OmpB*
